# Barriers and facilitators from the patients’ perspective in the follow-up of carpal tunnel syndrome and subacromial impingement syndrome: A qualitative study

**DOI:** 10.1371/journal.pone.0343222

**Published:** 2026-02-17

**Authors:** Eva Artigues-Barberà, Ester García-Martínez, José María Palacín Peruga, Marta Ortega-Bravo

**Affiliations:** 1 Department of Nursing and Physiotherapy. Faculty of Nursing and Physiotherapy, University of Lleida. Carrer de Montserrat Roig, Lleida, Spain; 2 Atenció Primària, Gerència Territorial de Lleida, Institut Català de la Salut. Rambla Ferran, Lleida, Spain; 3 Fundació Institut Universitari per a la recerca a l’Atenció Primària de Salut Jordi Gol i Gurina (IDIAPJGol). Gran Via de les Corts Catalanes, Barcelona, Spain; 4 Health Care Research Group, GRECS, Biomedical Research Institute of Lleida IRB-Lleida. Alcalde Rovira Roure, Lleida, Spain; 5 Onze de Setembre Primary Care Center, Gerència Territorial Lleida, Catalan Health Institute (ICS), Lleida, Spain; 6 Centre d’Atenció Primària Almacelles. Institut Català de la Salut. Melcior de Guàrdoa, Almacelles Lleida, Spain; 7 Department of Medicine. Faculty of Medicine, University of Lleida. Carrer de Montserrat Roig, Lleida, Spain; Universiti Malaya, MALAYSIA

## Abstract

**Introduction:**

Chronic musculoskeletal pain is one of the leading causes of disability worldwide, affecting 11–50% of the population. In Spain, it accounts for up to 50% of the chronic pain consultations conducted in primary care settings. The most common disorders are carpal tunnel and subacromial impingement syndromes, with treatments, including surgical and nonsurgical approaches, notably the use of ultrasound-guided injections to improve symptoms.

**Methods:**

A qualitative, phenomenological, and inductive study was conducted on patients diagnosed with carpal tunnel syndrome or subacromial impingement syndrome at primary care centers in Lleida to identify patient‑reported domains relevant to the routine follow-up of CTS and SAIS in primary healthcare, and to examine barriers and facilitators of treatments to inform patient‑centered follow-up protocols. Purposive sampling was used, and five focus groups were organised, selecting participants aged >18 years with or without prior surgical treatment. Data were collected between December 2022 and February 2023 using a semi-structured guide. The focus groups were recorded and thematically analysed using Atlas.ti.

**Results:**

Twenty-one patients aged between 44–75 years, predominantly women, participated in the study. The results were organised into six themes and 12 subthemes. The barriers identified were delays in diagnostic testing, overload of healthcare personnel, lack of coordination between care levels, and brevity of consultations. Effective communication, empathy of primary care professionals, and prompt management of treatments, such as injections, were highlighted as facilitators.

**Conclusion:**

This study highlighted the complexity of managing chronic musculoskeletal pain and the need for a multidimensional approach. The identified barriers, along with facilitators, such as effective communication and empathy of professionals, emphasize the relevance of strengthening problem-solving capacity in primary care and fostering better coordination between care levels. These findings suggest that addressing these aspects may support more tailored follow-up and contribute to improving patients’ experiences of care.

## 1. Introduction

Chronic musculoskeletal pain (CMP) is one of the leading causes of morbidity and disability worldwide, and carries a significant economic and social burden. Its prevalence varies between 11–50% in different populations according to international studies [1–3] In Spain, CMP has become the main reason for consultation for chronic pain in Primary Health Care (PHC), representing between 22–50% of general practitioners (GPs) visits and one third of all PHC consultations [2,4]. It is estimated that between 10–40% of the Spanish population experiences chronic pain caused by osteoarticular disorders, with higher prevalence in women and elderly individuals [2].

Although chronic low back pain is the most prevalent condition in the context of CMP disorder, there are other musculoskeletal pathologies that, while less prevalent, have a significant impact on the patients’ quality of life [5,6]. Among the most common are carpal tunnel syndrome (CTS) and subacromial impingement syndrome (SAIS) [7].

Carpal tunnel syndrome is the most common entrapment neuropathy, caused by compression of the median nerve within the carpal tunnel [8]. It is characterized by symptoms including paresthesia, dysesthesia, loss of sensation, and weakness, primarily affecting the thumb, index finger, middle finger, and the radial half of the ring finger [9]. While symptoms are typically localized to the hand, they may extend proximally to the forearm, upper arm, and even the shoulder [10]. CTS can be unilateral or bilateral, with studies showing that over 60% of patients have symptoms in both hands, and 80.70% exhibit bilateral nerve compression [11,12]. Its diagnosis is primarily clinical, and as reported in other countries such as the United Kingdom [13] and the United States [14], patients with CTS in Spain first seek care in primary healthcare and are treated at this level, representing a significant burden of visits [13].

On the other hand, SAIS is characterised by narrowing of the subacromial space with consequent injury to the bursa, tendon of the long head of the biceps, tendons of the rotator cuff, and coracoacromial ligament [15]. Symptoms include intense pain in the anterolateral aspect of the shoulder, particularly when raising the arm overhead, as well as weakness and a restricted range of motion [16,17], resulting in a loss of shoulder functionality [7]. There are no significant differences in prevalence between dominant and non-dominant shoulders, and sex-related differences remain inconclusive [18]. However, SAIS represents 44–65% of all shoulder pain consultations [7].

These conditions have been selected as representative in the context of PHC in this study for several reasons. CTS and SAIS are conditions that are usually managed autonomously by family doctors due to their well-defined diagnostic and clinical criteria. This makes both conditions particularly suitable for exploring the experiences of patients in PHC. Furthermore, in their therapeutic management, family physicians play an active role in administering treatments for pain relief such as medication and, in some cases, injections, as well as in organising follow-up visits and monitoring symptom evolution over time.

In the context of PHC, injections constitute a non-surgical therapeutic modality for the treatment of CTS and SAIS when other conservative treatments fail [19–21]. Corticosteroid or nonsteroidal anti-inflammatory drug injections, although minimally invasive, have been shown to improve symptoms and delay surgery due to their anti-inflammatory effects. However, despite their effectiveness in treating CMP [21,22], the potential side effects and/or complications of corticosteroids, such as tendon rupture, subcutaneous atrophy, changes in articular cartilage, and osteoporosis, limit their use [23,24]. Furthermore, because therapeutic outcomes are influenced by a precise injection technique [25], ultrasound has been used as a low-cost and easily accessible complementary procedure to physical examination and guide injections in PHC settings [26]. Two systematic reviews reported better outcomes in patients with shoulder pain after ultrasound-guided injection than those after unguided injection [27,28].

However, pain management due to its subjective, biopsychosocial, and multifactorial nature, demands a thorough individualised assessment [29]. Therefore, it is crucial to understand how patients experience CMP, its impact on their daily life, and their perspectives on the acceptability of proposed treatments. While current studies on CTS and SAIS in PHC primarily focus on clinical outcomes, less attention has been paid to how patients perceive the follow-up process itself, including aspects such as symptom monitoring, communication with healthcare professionals, adherence to recommendations, and acceptance of treatments. In this context, active patient involvement in monitoring their own condition, in collaboration with PHC professionals, may contribute to more patient-aligned follow-up strategies, particularly in chronic conditions requiring repeated assessments [30,31].

Despite this, there is limited evidence on which patient-reported domains should be systematically incorporated into follow-up protocols for CTS and SAIS in PHC, and how these domains could complement routine clinical assessment.

Building on the above, this qualitative study (Phase 1 of a two-phase project) aimed to identify patient-reported domains relevant to the routine follow-up of CTS and SAIS in primary healthcare (such as pain impact, functional limitations, self-management strategies, adherence, and communication), and to examine perceived barriers and facilitators of treatments to inform patient-centered follow-up protocols.

The insights gained from this first phase will inform the design and monitoring of Phase 2, a prospective observational study with medications to be conducted in PHC centers in Lleida. Specifically, the patient-reported domains identified in Phase 1 will be operationalised into a structured follow-up questionnaire incorporated into the data collection notebook for Phase 2. This second phase will evaluate functional outcomes and overall satisfaction in patients with CTS or SAIS undergoing three treatment approaches: corticosteroid infiltration, ultrasound-guided corticosteroid infiltration, and conventional oral pharmacological treatment combined with joint rest. Follow-up is defined as both scheduled post-treatment (infiltration) visits and patient-reported interactions with primary care professionals, including face-to-face and remote consultations (telephone or online). This sequential approach seeks to ensure that follow-up protocols reflect patient priorities and experiences, without presuming direct effects on clinical outcomes beyond those empirically assessed. Ultimately, the results of this study are expected to contribute to a more holistic and person-centred approach to the follow-up of CTS and SAIS in primary healthcare, by better aligning monitoring processes with patients’ experiences and priorities.

## 2. Methods

### 2.1. Design

A qualitative study was conducted using a phenomenological approach with an inductive reasoning process to explore participants’ lived experiences and perceptions, allowing for a deeper understanding of the studied phenomenon [32]. This design seeks to identify commonalities in how individuals perceive and interpret a phenomenon, aiming to distill their experiences into a universal essence [32]. The protocol of this study has been previously published [33].

### 2.2. Recruitment and participants

The recruitment period lasted from 1 December 2022–28 February 2023. Purposive sampling [34] was used to recruit adults with CTS or SAIS from PHC Centres (PHCCs) of Lleida, which is part of the Catalan Public Health System.

Inclusion criteria were as follows: 1) patients diagnosed with CTS or SAIS; 2) women and men aged ≥18 years; and 3) those who had or had not previously received surgical treatment of the area. Patients with cognitive impairment, as well as those who did not speak Spanish or Catalan fluently, were excluded.

Participants were recruited by three GPs from PHCCs in Lleida. Initially, we collected patient data from the electronic medical records stored in the E-CAP computerised database of PHC medical history from the Catalan Health Institute. Subsequently, the GPs contacted potential participants by phone to arrange an in-person visit. During this visit, they invited individuals to participate in the study, provided them with a written informed consent form and a participant information sheet, and explained the study’s objectives and procedures in detail. Written informed consent was obtained from all participants during this encounter, prior to the collection of any data or scheduling of focus group discussions. Contact details were collected only after participants had signed the IC form, in order to organise their participation in the focus groups.

Forty-four patients diagnosed with either CTS or SAIS from five PHCCs were contacted, but 17 declined to participate. Five focus groups were organised based on participant profiles, availability, and preferences, ensuring group heterogeneity by considering sex, age, diagnosis, and sociocultural status. However, three did not attend the focus group without providing any reason, two were excluded due to health issues, and one withdrew written informed consent during the session. Finally, 21 patients, eight with CTS and 13 with SAIS, mostly women (52.38%), aged between 44–75 years, participated in this study (Table 1).

**Table 1:** Participants’ demographics (n = 21).

CMP condition	ID code	Sex	Age	PHCC	Focus Group
**Carpal tunnel syndrome**	CTS 1	Female	55	Alcarràs	GF1
	CTS 2	Female	87	Eixample	GF2
	CTS 3	Female	35	Bordeta-Magraners	GF2
	CTS 4	Female	59	Onze de Setembre	GF2
	CTS 5	Male	69	Balàfia-Pardinyes-Secà	GF3
	CTS 6	Male	71	Eixample	GF3
	CTS 7	Male	58	Eixample	GF3
	CTS 8	Female	64	Bordeta-Magraners	GF4
**Subacromial impingement syndrome**	SAIS 9	Female	68	Onze de Setembre	GF1
	SAIS 10	Female	72	Onze de Setembre	GF1
	SAIS 11	Male	48	Bordeta-Magraners	GF1
	SAIS 12	Male	63	Bordeta-Magraners	GF1
	SAIS 13	Female	61	Onze de Setembre	GF2
	SAIS 14	Male	76	Onze de Setembre	GF3
	SAIS 15	Male	50	Alcarràs	GF3
	SAIS 16	Female	68	Bordeta-Magraners	GF4
	SAIS 17	Female	52	Eixample	GF4
	SAIS 17	Female	79	Eixample	GF4
	SAIS 19	Male	58	Bordeta-Magraners	GF5
	SAIS 20	Male	57	Eixample	GF5
	SAIS 21	Male	41	Eixample	GF5

### 2.3. Data collection

Data collection was conducted by MOB and EAB between December 2022 and February 2023 using the focus groups as a technique. EAB moderated the group and MOB supervised the participants’ interactions and took notes, which helped interpret and understand the discourses during the analysis.

An open-ended question was used as a guide to identify variables that could be introduced during the follow-up of CMP (See S1). A semi-structured guide was developed based on scientific literature and previous clinical experience of researchers. The focus groups were flexible, enabling the exploration of new avenues that emerged spontaneously, and providing the opportunity to delve into topics that were not initially considered [35]. All the focus groups took place in a designated room at the PHCC, easily accessible to participants, and were intimate and comfortable for smooth communication without interruptions, interference, or noise. The focus groups were video-recorded and lasted for approximately 90 min. Focus groups continued until thematic saturation was reached [32]. Finally, all the data were anonymised, with an alphanumeric code assigned to each participant and focus group, and transcribed textually for analysis.

### 2.4. Data analysis

All focus groups were transcribed textually, reviewed, and imported into the Atlas.ti 9. An inductive thematic analysis was conducted by EAB and EGM. Inductive and deductive coding were performed to identify the meaning units of the text.

To ensure inter-coder reliability, two researchers (EAB and EGM) and one external (CCS) independently coded the interview transcripts. The initial codes were compared, and discrepancies were discussed and resolved through consensus during team meetings. This process ensured consistency in coding and refined the coding framework. After the initial agreement, the refined framework was applied to the remaining transcripts.

All the interview transcripts were thoroughly read, and each sentence or paragraph with the same meaning was assigned a code. The codes were then grouped into subthemes and main themes [36].

### 2.5. Ethical considerations

All the participants were informed that participation was voluntary and that no financial rewards would be given, based on which they all provided written informed consent. Information regarding the project was provided, and participants were given the opportunity to express their concerns or ask any questions pertaining to the study. Data confidentiality was assured according to Regulation (EU) 2016/679 of the European Parliament and Council of 27 April 2016. This study followed the principles of the Declaration of Helsinki and Good Clinical Practice Guidelines. This study was approved by the Clinical Research Ethics Committee (BLINDED FOR PEER REVIEW) with the code (BLINDED FOR PEER REVIEW).

### 2.6. Rigour

The rigour of the study was ensured by following the Consolidated Criteria for Reporting Qualitative Research guidelines [37] and applying the trustworthiness criteria for qualitative studies following Guba and Lincoln [38]. To achieve reliability, the entire process is documented and detailed in the Methodology section. To enhance credibility, the analysis was triangulated using the CCS as an independent researcher. The confirmation of the results was ensured by including direct quotes from participants supporting the interpretation of the findings, and the entire workflow was overseen by different team members who were not directly involved in the analysis of the results.

## 3. Results

The results were organized into six themes and 12 subthemes. The most representative patient quotations are provided in italics in this section. See Table 2 for the main themes, subthemes, and most representative quotes for quick reference.

**Table 2:** Themes, subthemes and most representative quotes.

**Themes**	**Subthemes**	**Quotes**
**Healthcare System and Waiting Lists: Significant Room for Improvement**	*Waiting Lists*	“When you’re in pain and can’t sleep, what happens is you get desperate. You say, ‘When will it be my turn?’” (SAIS 14)
	*Healthcare System*	“It’s not your problem. The problem is that there’s a lack of staff. Lack of facilities to do rehab (…) there comes a point at 8 in the morning when you’re there, at the 11th of September (PHCC), and it feels like the world is ending.” (SAIS 11)
	Continuity and Coordination between Levels of Care	“There’s a lack of communication between them (PHC and PH). My specialist told me, ‘You won’t need to come anymore. If you want to come, feel free, just ask your PHC doctor, and he’ll refer you here (hospital)... The PHC doctor is the one who knows the patient.” (SAIS 10)
**Follow-Up after Injection: an Essential Process that must be Individualised**	*Questions and General Aspects*	“It’s been three or four weeks since I had the injection... What would I like them to do now? Maybe, I don’t know, for us to check again to see how I’m doing, whether I’ve recovered or not...” (SAIS 14)
	*Professional Conducting the Follow-Up*	“The follow-up should be done by the professional who injectioned us.” (SAIS 17)
	*Type and Frequency of Follow-Up*	“A follow-up call would be a good way to ask if I’ve noticed any improvement with the treatment.” (SAIS 11)
**Expectations, Experiences, Perceived Benefits, and Effectiveness of Injections**	*Expectations and Experiences Relating to Injections*	“It caused me a lot of distress... And it’s true that on the day I went, the doctor did it really well (the injection), and I didn’t feel a thing. It was five minutes.” (CTS 1)
	*Perceived Benefits of Injections*	“It’s true that once you’ve had the injection, that feeling of being disabled is no longer there. You can start doing things.” (SAIS 11)
	*Perceived Effects after Injection (Perceived Effectiveness)*	“A week after the injection, it seemed to be going well. I was pain-free for a month, but then it came back step by step.” (SAIS 19)
**Physiotherapy: A Key Element in the Treatment Process**		“If treatment is undertaken with medication but rehabilitation is not conducted afterwards, no progress is made” (SAIS 11) “It’s important that you’re supervised so that the rehab is effective.” (SAIS 15)
**Considerations Regarding Other Pharmacological Treatments**		“Taking pills isn’t enough; it takes away the pain one day, but it’s not the solution.” (SAIS 20) “Since the operation, I stopped going to the gym... I feel more useless when I exercise because there are things I struggle with (my shoulder).” (CTS 2)
**Receiving Information and the Therapeutic Relationship: a Perceived Need**	*Received Information*	“A doctor comes to you and says one thing, and another doctor who isn’t yours says something about the tests that the other hasn’t mentioned... They take it into account, but they don’t tell you because they don’t want to.” (CTS 5)
	*Need for Information*	“It is not a justification to say that the patient cannot be well informed because there is no time” (SAIS 11)
	*Therapeutic Relationship*	“Lack of confidence, and always “ME”, not as much as before, because now there are many youngsters, but the old doctors… “A tall order”. I think that going through circumstances like going to the traumatologist, “the ancient doctors’” word was law.” (SAIS 10)

### 3.1. Theme 1. Healthcare System and Waiting Lists: Significant Room for Improvement

#### 3.1.1. Waiting lists.

There is widespread concern regarding waiting lists, particularly those related to access to diagnostic imaging tests and rehabilitation services, which cause desperation among patients experiencing intense pain. The participants highlighted instances in which the slow pace of the system in scheduling appointments for rehabilitation services and pain management units led them to seek care through a private healthcare system.

*“When you’re in pain and can’t sleep, what happens is you get desperate. You say, ‘When will it be my turn?’.” (SAIS 14)*

*“For this to go away, you either have to wait nearly two months before you can start rehab or go private... They told me when I was already healed.” (SAIS 11)*

The delay in accessing rehabilitation was associated with a lengthy referral process that began in the PHC, moved to hospital care (HC), and ultimately resulted in referral to the respective service.

*“Access should be quicker so you can do rehab with a physiotherapist... These are the steps, the doctor told me: ultrasound, injection, and then maybe rehabilitation.” (SAIS 20)*

However, the perceptions of the care received in PHC were positive, as the treatments offered at this level of care were managed more swiftly and effectively. The participants reported that the process of obtaining appointments for injections typically took no longer than a week. In some cases, injections were administered on the same day as the consultation in the PHC, which facilitated more rapid pain management, significantly reduced pain and provided quicker recovery.

*“I went to the PHCC and asked my doctor ‘What can we do? Can I do rehab?’ And she said, ‘Well! Rehab…’ I said, ‘Injections? And she goes, ‘Well, wait, I’ll ask the doctor (who does the injections).’ She told me, ‘Today’s Friday, I think the doctor isn’t here, but don’t worry, he’ll call you.’ They called me a week later, gave me an appointment, and that was quick. But follow-up? None at all, eh!” (CTS 4)*

#### 3.1.2. Healthcare system.

The participants highlighted the evident limitation of human resources, which, in their view, imposed an excessive workload on healthcare staff and hinders their ability to provide adequate care, leading to the perception of a collapsed system. The lack of healthcare personnel is also associated with accessibility issues in medical care, as evidenced by the long waiting lists. The lack of adequate facilities further exacerbates this problem, as it hampers the effective implementation of treatments, particularly in rehabilitation.

*“It’s not your problem. The problem is that there’s a lack of staff. Lack of facilities to do rehab (…) there comes a point at 8 in the morning when you’re there, at the 11th of September (PHCC), and it feels like the world is ending.” (SAIS 11)*

*“I could agree with what he says a hundred percent, that the problem is, alongside the desk, there are fewer and fewer of you, and on the other side, we’re more and more… the problem for me is that there are not enough doctors and nurses. The (professionals) that are here have treated me well, and it works.” (SAIS 14)*

Another aspect mentioned was the brevity of consultations, which hindered professional-patient communication and posed a barrier to receiving information about their health condition and available treatments. Moreover, it was generally reported that in the past, the perception of time was more relaxed, and patients felt they were listened to.

*“Doctors used to be like a confessional. There were many conditions that weren’t just medical; there was perhaps more of an emotional-psychological side. And before, they listened because there was a more relaxed system of time.” (SAIS 13)*

#### 3.1.3. Continuity and coordination between levels of care.

Coordination among various levels of care has emerged as a critical area for improvement. Coordinated care, as perceived by patients, refers to the communication and collaboration between professionals across different levels of care, aligned with the organizational circuits that structure the interaction between primary and hospital care in the Spanish health system. Participants noted that the lack of teamwork between professionals in PHC and HC created a disconnect that resulted in contradictory recommendations and feelings of starting over when changing the levels of care. Furthermore, the lack of trust in the assessment of PHC doctors by HC professionals directly affected patients’ confidence in the care process and treatments received. The participants called for improved communication between these levels of care.

*“There’s a lack of communication between them (PHC and PH). My specialist told me, ‘You won’t need to come anymore. If you want to come, feel free, just ask your PHC doctor, and he’ll refer you here (hospital)... The PHC doctor is the one who knows the patient. When you go there (hospital), if the PHC doctor has done his job well, they already have the process started, and you don’t need to go back to your PHC doctor. I’ve been seen by two doctors. For whatever reason, they also get sick; maybe they were off sick or unwell, so another doctor attended to me. This means starting over.” (SAIS 10)*

### 3.2. Theme 2. Follow-Up after Injection: an Essential Process that must be Individualised

#### 3.2.1. Questions and general aspects.

The participants highlighted the importance of conducting an individualised assessment during follow-up, addressing aspects related to the effectiveness of the injection in improving their symptoms and potential side effects of the treatment. Other relevant aspects considered during follow-up included functionality, pain intensity, and sleep quality. The general discourse indicated that follow-up after receiving treatment via injection was perceived as necessary by the participants, providing them with reassurance and confidence in knowing that someone is caring for them. Only one participant stated that they felt it was acceptable for no follow-up to be conducted after injection.

*“It’s been three or four weeks since I had the injection... What would I like them to do now? Maybe, I don’t know, for us to check again to see how I’m doing, whether I’ve recovered or not, or if I could recover more, or, well, look, this is left over or that, and you could do this; a clear treatment plan that I need to follow from now on.” (SAIS 14)*

*“The fact that someone asks you if you’ve noticed any improvement, how you’re feeling, if the medication has worked well for you, if you had a good night, or if you managed to sleep today... with just four questions, it gives you reassurance. And if there’s someone on the other side who cares about how you are, it gives you confidence.” (SAIS 11)*

#### 3.2.2. Professional Conducting the Follow-Up.

The participants expressed indifference regarding whether the follow-up was carried out by doctors or nurses; however, they preferred that the follow-up be conducted by the same professional who performed the injection or was present during the process.

*“The follow-up should be done by the professional who injectioned us.” (SAIS 17)*

*“Now there’s the resident... and the nurse, because, the doctor who did the injection, no! (...) well, the professionals who were there at that time to carry out the treatment.” (CTS 4)*

#### 3.2.3. Type and Frequency of Follow-Up.

Regarding the type of follow-up, the majority suggested a phone call and accepted it if face-to-face follow-up was impossible.

*“A follow-up call would be a good way to ask if I’ve noticed any improvement with the treatment.” (SAIS 11)*

*“If they had called me, I would have said: ‘Excuse me, can I have an appointment? Though a phone call would have been enough.” (CTS 4)*

*“I yes, I would appreciate a follow-up call.” (SAIS 17)*

As for the frequency of the follow-up, this was an aspect that prompted differing viewpoints. While some of the participants believed that the frequency should be adapted to the severity of each case, others suggested intervals of fortnight or even a month. The importance of making the first follow-up call at strategic moments was emphasised, considering the peak effects of the injection to more accurately assess the effectiveness of the treatment.

*“For me, since it’s gone well and I’m not scared, I suppose that with just a couple of calls during the process, I think it could work well for me. But there are people who are more anxious and might need more calls. I think a couple of times is enough because you already notice a lot of improvement.” (SAIS 10)*

*“Well, it’ll depend on the severity of each case. With my case, they’d check on me once every fortnight or once a month. ‘How’s this been? How are you feeling? How’s the rehab going?’ That would be more than enough.” (SAIS 11)*

Additionally, there were cases in which ultrasound was suggested one month or a month and a half after the injection for a more thorough evaluation of treatment effects.

*“They did the injection, and it’s been a month or a month and a half, and if I’ve had an injection, they should do another echography—this cost nothing!” (SAIS 14)*

### 3.3. Theme 3. Expectations, Experiences, Perceived Benefits, and Effectiveness of Injections

#### 3.3.1. Expectations and Experiences Relating to Injections.

The participants expressed diverse expectations that reflected their attitudes towards the injection process or their prior beliefs about the treatment. However, experiences related to the injection procedure were generally positive.

Some of the participants reported feelings of distress, fear, and emotional tension before the injection process; however, their actual experiences were positive. This highlights the contrast between initial negative expectations and reality of the procedure, questioning the need for pre-procedural anxiety.

*“It caused me a lot of distress... And it’s true that on the day I went, the doctor did it really well (the injection), and I didn’t feel a thing. It was five minutes. I even told the doctor, ‘What a fool! Why did I suffer so much if I didn’t feel a thing?” (CTS 1)*

In line with this, repetition of the procedure can provide a sense of familiarity, reducing the pre-existing emotional tension associated with the unknown: *“you know where you’re going for the second injection”*. Other participants approached the injection with resignation, considering it *“a necessary evil” (SAIS 13)*.

The results revealed that having negative expectations before the procedure led some participants to decide against undergoing injection, arguing that *“I’m convinced the injection won’t bring me anything good” (SAIS 20)*. Other reasons for not undergoing the procedure included beliefs about whether the treatment truly addressed the underlying cause of the pathology.

*“The injection masks the pain. Because you feel fine, you go to work and end up aggravating it (your shoulder) because the pain is hidden, and it doesn’t hurt.” (SAIS 19)*

Conversely, some participants approached the injection with a positive attitude, seeking relief from pain or thinking, *“tonight I’ll be able to sleep well” (SAIS 13)*. One participant recounted, *“I went for the injection as if I were going to a party because I was seeking relief” (CTS 4)*.

#### 3.3.2. Perceived Benefits of Injections.

The participants’ discourse revealed a general perception of the benefits of injections as an effective treatment for relieving pain both during the day and night, thereby promoting high-quality sleep. Pain relief also enabled them to engage in the necessary rehabilitation therapies and exercises to regain functionality, which in some cases is perceived as a curative treatment.

*“The injection, as far as I understand, is to get rid of the pain, but what cured me was the rehab.” (SAIS 10)*

The participants highlighted how undergoing this procedure allowed them to carry out daily activities, contributing to minimising feelings of disability and providing a sense of well-being and normality.

*“It’s true that once you’ve had the injection, that feeling of being disabled is no longer there. You can start doing things.” (SAIS 11)*

These perceived benefits, along with the satisfaction with the treatment received from healthcare professionals in PHC, emphasise the positive aspects of receiving treatment at this level of care, as *“it’s a bit more familiar and gives you more confidence” (SAIS 19)*.

However, some participants shared less positive views on the injection, feeling that the beneficial effects on pain were minimal and did not contribute to improving other symptoms, such as tingling or fading quickly over time. It was also reported that *“the first injection went well for me, but I didn’t notice as much improvement with the second” (SAIS 13)*.

#### 3.3.3. Perceived Effects after Injection (Perceived Effectiveness).

Opinions vary widely regarding treatment through injection. Although some of the participants claimed that, after a long period of other treatments, the injection provided them with real pain relief, others asserted that it was also a therapeutic approach that was ineffective and short-lived. In relation to the latter, the accounts revealed that they produced few beneficial effects, that these effects were very limited in duration, or that, although they provided slight relief from pain, they did not contribute to improving functionality. Furthermore, the participants perceived that the second injection was not as effective as the first. Regarding side effects, a *“rebound effect” (CTS 4)* was reported after the injection, whereby pain increased after receiving this treatment, but improved over the following days:

*“I did a lot of things and wasn’t getting anywhere. I was taking loads of pills… And there was no way forward. That’s when we reached the conclusion with the doctor to go for the injection.” (SAIS 10)*

*“A week after the injection, it seemed to be going well. I was pain-free for a month, but then it came back step by step.” (SAIS 19)*

*“When they give you the injection, the pain goes straight away (...) After, I had a terrible night with excruciating pain. An awful pain! I’d never experienced anything like it. But after a few days, it started to go away.” (SAIS 16)*

### 3.4. Theme 4. Physiotherapy: A Key Element in the Treatment Process

The participants’ perceptions highlight the role of rehabilitation in the treatment process, as *“if treatment is undertaken with medication but rehabilitation is not conducted afterwards, no progress is made” (SAIS 11)*. Therefore, they noted the beneficial aspects of rehabilitation, including pain relief and improved mobility. Some of the participants preferred this approach over pharmacological treatments.

However, the results revealed a clear influence on the participants’ perceptions of the benefits of rehabilitation based on the professional prescribing it and, above all, the availability of supervision while performing exercises. Generally, GP provide only home-based exercises, and there is a belief that specific questions regarding the correct execution of these exercises need to be addressed. This generated a fear that performing the exercises might exacerbate pain.

*“I do aquagym. But sometimes I ask the doctor because I think, I don’t know if I’m doing it right or wrong, because I always go for a walk as well... for the problem I have. So I ask the doctor, ‘Is it okay to exercise? Is it not okay? Am I doing it right or wrong?’ And the doctor doesn’t know how to answer me.” (CTS 1)*

*“There are times when I can’t do the exercises on the sheet because it causes me more pain.” (SAIS 18)*

Thus, the physiotherapist’s role was perceived as ideal for carrying out rehabilitation treatments with supervision during exercise because a lack of guidance can lead to counterproductive outcomes.

*“It’s important that you’re supervised so that the rehab is effective.” (SAIS 15)*

The participants emphasised the role of re-education within the context of physiotherapy, considering it a key tool for adapting to illness in daily life and work demands. When combined with rehabilitation, this offers a comprehensive strategy for addressing such adaptations. The physiotherapist’s role was deemed crucial in providing personalised techniques and recommendations for implementing both rehabilitation and re-education.

*“What would be interesting is the re-education of the patient (...) guidance for the patient regarding their condition, how they should modify things while they don’t have treatment, whether it’s surgery or injections (...) The physiotherapist is the one who knows all the peculiarities and bodily pains.” (SAIS 13)*

However, two participants shared the perception that physiotherapy is not a definitive treatment for pain, and that exercises cannot be performed when experiencing pain.

*“I did rehab twice, and I improved quite a bit. That lasted six years, but now I’m having problems with my shoulder again.” (SAIS 20)*

*“And it doesn’t work with pain. The doctor told me, if you’re in pain, don’t do it; you can’t do it with pain.” (SAIS 21)*

On many occasions, the participants reported that they had to cover the cost of physiotherapy treatment themselves and attend private clinics, either because they had never been referred to public healthcare or because the number of prescribed sessions was insufficient. This poses a barrier to accessing rehabilitation treatments, forcing patients to incur costs that they cannot afford in many cases.

*“I think it would be more worthwhile to go to a physiotherapist... Then when you ask for help, they tell you, ‘go private.’ I go.” (SAIS 18)*

*“I’d also go (to the physiotherapist), and if necessary, I’d find the money from wherever and go. But now I just take a pill and get on with it... But I think it would do me good every now and then.” (SAIS 16)*

#### 3.5. Theme 5. Considerations Regarding Other Pharmacological Treatments.

The participants perceived oral pharmacological treatments as insufficient for improving pain; moreover, they produce side effects. These effects include increased blood pressure, worsening diabetes, and digestive problems. Therefore, oral pharmacological treatments were perceived to have a limited effect over time, and taking them was seen as posing more risks than benefits.

*“Taking pills isn’t enough; it takes away the pain one day, but it’s not the solution.” (SAIS 20)*

Another relevant aspect that emerged was the potential barrier to accessing medication. While some participants reported never having difficulty obtaining the recommended medication, others reported that their medication was occasionally unavailable at the pharmacy. In contrast, the participants shared the feeling that having to take so much medication or *“having to carry naproxen in my pocket all day” (SAIS 19)* was a barrier to effectively managing their condition.

Regarding surgical treatment, the participants generally expressed concern or fear about undergoing this procedure.

*“I didn’t want to have surgery, even though most people say they’ve come out fine. But I think... I’ve got time to have the surgery.” (CTS 4)*

*“Because if they tell me, ‘I’m going to operate on you’, I wonder, will I be able to move the way I do now, even if it hurts? I don’t know! So then...” (CTS 5)*

However, one participant decided to proceed with surgical treatment and reported that the effects were detrimental, limiting their mobility and leading to feelings of uselessness.

*“Since the operation, I stopped going to the gym... I feel more useless when I exercise because there are things I struggle with (my shoulder).” (CTS 2)*

### 3.6. Theme 6: Receiving Information and the Therapeutic Relationship: a Perceived Need

#### 3.6.1 *Received Information.*

The accounts from the participants revealed significant variability in the information provided by PHC professionals, which, according to their perceptions, seems to indicate that each doctor provides information based on what they consider important.

*“A doctor comes to you and says one thing, and another doctor who isn’t yours says something about the tests that the other hasn’t mentioned... They take it into account, but they don’t tell you because they don’t want to.” (CTS 5)*

With respect to information related to the underlying cause of their condition, the participants indicated receiving very diverse explanations, such as having inflammation, having possibly experienced tendon inflammation in the past, and *“it will happen again” (SAIS 16)*, that their pain and movement limitations are due to calcification, or even that it is wear and tear from their work.

Regarding treatment, specifically injections, the information received was homogeneous in terms of timing, as they reported that professionals informed them that the injection could be repeated every three to four months. Regarding the injection procedure, some participants reported not receiving any information, whereas others stated that the doctor had explained the procedure to them. In contrast, it was evident that at times doctors or acquaintances of patients send messages that are less than optimistic regarding the effectiveness of injection or the durability of its effects, which in some cases led to *“fear of poor results”* (SAIS 20).

*“The doctor only explained to me that the injection was to remove the pain.” (SAIS 15)*

*“He said, ‘I’m going to give you an injection that will hurt a bit…’ And with the ultrasound, he was explaining it to me. ‘Look, it’s the tendon. I’m going to injection here.’” (SAIS 17)*

Concerning rehabilitation or exercises involving the shoulder joint, some messages received from GPs appeared negative, with some professionals not recommending physiotherapy. Some even advised the participants to move their affected limbs. Additionally, discouraging messages are conveyed through rehabilitation services.

*“The doctor told me not to lift weight, not to do anything with my arm.” (SAIS 18)*

*“When I went about my arm, it was already destroyed. I went for rehab after two months, and they told me I was late.” (SAIS 14)*

#### 3.6.2. Need for Information.

Although the general discourse of the participants pointed out that receiving information about their condition and treatment options was essential, they also emphasised that this information should be individualised. Although some participants reported a need for complete and detailed information, others argued that they might feel overwhelmed by receiving too much information. The former group asserted that receiving all the information, both positive and negative, provided reassurance and facilitated the development of self-management strategies, allowing them to feel part of the process and make informed decisions. Their perception was that receiving partial information was worse than receiving no information, as it encouraged ruminative thoughts. One participant stated that *“It is not a justification to say that the patient cannot be well informed because there is no time”* (SAIS 11).

*“If for example, some pills can cause dizziness and they don’t tell you, you take the pill and later think, what’s happening to me? If you know it can cause dizziness, you understand that’s what it is.” (SAIS 13)*

*“Because of the lack of knowledge of what it means and what can happen. Whether it can go well or not. I would lack information.” (CTS 3)*

This lack of information led the participants to seek it on their own, with the Internet as their primary source of information. They also reported asking a physiotherapist, who is apparently a more accessible professional, to resolve their doubts.

*“Google is wonderful, isn’t it? [...] Well, due to the lack of information, since they don’t provide it, you look for it.” (SAIS 13)*

*“During the waiting time for the injection, I looked for information. Then I went to talk to my physiotherapist.” (SAIS 15)*

In terms of the format for delivering information, although the importance of receiving verbal information was highlighted, participants also expressed that it was difficult to retain it. Therefore, written information, specifically on paper, was rated very positively for convenience and ease of reference.

#### 3.6.3. Therapeutic Relationship.

Overall, the patients expressed satisfaction with the treatment received from professionals, particularly in PHC settings, where the GP knew the person and their environment, thereby generating trust and a sense of support. Furthermore, there was a perception of an evolution in patient treatment compared to previous times. The participants expressed that greater effort is now being made to understand the underlying causes of conditions, and that a new generation of professionals is less imperative, making them feel more supported.

*“Lack of confidence, and always “ME”, not as much as before, because now there are many youngsters, but the old doctors… “A tall order”. I think that going through circumstances like going to the traumatologist, “the ancient doctors’” word was law.” (SAIS 10)*

There have also been reports on how the e-consultation tool facilitates communication with professionals and are useful for resolving doubts and addressing issues that do not require appointments.

*“With my GP, we sometimes exchange messages through the e-consult. And he said to me, ‘If you need anything, just write to me.” (SAIS 15)*

## 4. Discussion

This qualitative study aimed to identify patient‑reported domains relevant to the routine follow-up of CTS and SAIS in primary healthcare, and to examine barriers and facilitators of treatments to inform patient‑centered follow-up protocols.

A widespread concern among the participants was the delay in access to diagnostic imaging tests and rehabilitation services, which generated frustration among patients experiencing intense pain. This finding is consistent with previous studies that identified waiting lists as a significant barrier to the management of chronic pain [39,40]. For exemple, Lynch et al. [40] reported that such delays may be partly explained by factors, including limited access to publicly funded healthcare professionals (e.g., physiotherapists), inadequate remuneration for the additional time required to treat complex chronic pain, and a lack of training in pain management among some professionals. While our participants did not explicitly mention a lack of knowledge among healthcare providers, some reported seeking private rehabilitation services due to delays in the public system. These accounts underscore the perception of a need to optimise workflows and reduce waiting times. Furthermore, a systematic review demonstrated that waiting for as little as five weeks for care was associated with a deterioration in health-related quality of life among patients with chronic pain, while a reduction in waiting times was linked to greater satisfaction [40] and a more active use of pain self-management strategies [41].

The limitations of human resources and burden on healthcare staff were also noted as factors affecting the quality of healthcare, contributing to the perception of a healthcare system widely documented to be overwhelmed following the COVID-19 pandemic. Many countries have implemented telemedicine tools to address these structural issues. Online platforms, email, and e-consultations are part of asynchronous telemedicine and are used by patients and professionals to manage appointments, make diagnoses, and even prescribe medications [42]. E-consultation has been proposed to reduce waiting times for chronic pain care [39], address enquiries by decreasing user travel time, and facilitate the self-management of cases and professional schedules. E-consultation is an asynchronous teleconsultation service integrated into public healthcare information systems, operational in Catalonia in late 2015 in PHCCs [43]. The use of telemedicine has increased rapidly since the pandemic in March 2020 [42], and continues to increase. In the present study, e-consultation emerged as a tool that facilitates communication between users and professionals, proving useful in addressing questions and handling issues that do not require scheduled appointments. Despite this potential and the benefits of improving access and efficiency in consultations, a qualitative study conducted in England among GPs reported that most electronic consultations necessitated scheduling a phone or in-person appointment because the consultation lacked sufficient information for clinical decision-making, further increasing the clinical workload of professionals [44], along with administrative burdens and barriers to efficient workflows, such as poor usability [42]. Despite the inconsistencies in telemedicine reported by Leighton et al. [42], the implementation of strategies and policies that increase human resources and improve consultation time management could help address these barriers and advancing the development of a comprehensive and continuous care model.

Another barrier is the lack of continuity and coordination between PHC and specialised healthcare (SHC), leading to contradictory recommendations and the need to restart care processes, negatively affecting trust in the healthcare system. This finding is consistent with previous qualitative studies revealing that joint treatment plans involving PHC and SHC for patients with chronic pain are rarely established, despite their importance and necessity [45,46]. Ricci-Cabello et al. [47] indicated that trust is an essential factor for patient safety, built through continuity of care and a patient-centred approach. Improving communication among professionals across different care levels is the key to addressing this disconnection. Various studies advocate for an integrated and coordinated approach to managing CMP, emphasising that the quality of communication between health providers and between healthcare professionals and patients enhances patients’ trust in their providers, increases their satisfaction with treatment [48], and, in turn, leads to improved adherence, better health outcomes [49,50], and greater satisfaction with healthcare services [51].

The brevity of medical consultations has been identified as a challenge hindering effective communication between professionals and patients, preventing the provision of sufficient information about health conditions and available treatments. This lack of information, coupled with the variability in information provided by professionals regarding pathology and its treatments, has emerged as a significant barrier that has led many patients to seek information independently, primarily online, thereby increasing uncertainty and emotional distress, which could be partially attributed to information overload [52]. This communication problem aligns with studies suggesting that many errors reported by patients are related to deficiencies in the communication process and interpersonal skills of professionals [47]. Sharkiya [53] proposed enhancing effective and high-quality communication by complementing various strategies related to nonverbal communication with verbal communication, planning communication, and setting outcomes focused on the individuality of the patient. Effective communication was highlighted by the participants as a facilitator of the process.

Furthermore, the participants emphasised the need to gain a deeper understanding of injection procedures, their effects and adverse outcomes, durability, frequency, pain self-management advice, and complications following injection. Physician–patient communication has been the subject of study since ancient times [54]. Ensuring a clear and detailed exchange is essential not only for patients to feel well informed but also for them to actively participate in decision-making regarding their treatment. The benefits of participation have been documented, compelling many Western countries to promote patient involvement and discuss the assigned position of passivity as the default form of communication [55]. Shared decision-making requires a unified effort not only from professionals but also from organisations that wish to overcome the barriers to its development in routine clinical practice [56].

Despite these difficulties, the participants highlighted the therapeutic relationship with PHC professionals as facilitators, emphasising their closeness and empathy, which helped mitigate the lack of information received, as well as the swift and effective management of treatments, such as injection, many of which were performed on the same day as the consultation. According to Soler-Pérez et al. [57], the ability to perform injections in a PHC centre saves time and suffering for the patient, accelerates functional recovery, and avoids unnecessary referrals and waiting times. Additionally, GPs from PHC benefit from this approach, as local injection enhances their limited resources, improves their problem-solving capacity, and strengthens their relationship with patients [57].

However, the participants stressed the importance of individualised follow-ups after injection, providing them with reassurance and confidence. Follow-up should assess treatment effectiveness, possible side effects, sleep quality, functionality, and pain intensity. The aspects mentioned by patients have been considered key indicators for evaluating health outcomes in patients with non-oncological chronic pain [58]. The preference for telephone calls over in-person follow-ups exhibited considerable variability in patients’ needs and preferences, reinforcing the importance of personalising the follow-up approach for chronic pain management, as suggested by previous studies [59,60]. Furthermore, the individualisation of follow-up was highlighted as a facilitator by the participants in the study. In line with the observations of Turk and Okifuji [59], the treatment of chronic pain should not be understood as an acute problem, but requires ongoing attention and prolonged follow-up over time, as patients often require more complex adaptations. In this vein, Berrocoso Martínez et al. [60] recommended implementing a follow-up plan that included scheduled visits at 48–72 h, either by telephone or telematics, and an in-person consultation after 15 days. Once treatment is established, reviews should be spaced one month, three months, and six months apart to adjust the treatment and ensure improvement of the patient’s functional capacity. Other authors, such as Tristancho et al. [58], have also highlighted the importance of reaching a consensus on therapeutic interventions for patients considering their preferences and expectations.

In this study, the expectations and experiences of the participants regarding injections varied considerably. Although most participants expressed positive expectations, some voiced negative expectations regarding outcomes, leading them to reject treatment. This decision can be explained within the framework of the fear-avoidance model, which posits that negative expectations and fear of pain lead to fear of action [61]. However, familiarity with the procedure, acquired through repeated injection, helped reduce emotional tension by alleviating initial fear. Therefore, following the injection, participants generally described perceived benefits not only in terms of symptom relief but also in relation to greater independence in daily activities and participation in rehabilitation. These perceptions align with the results of quantitative studies conducted on painful shoulder pathology, whose findings demonstrated that after a single joint injection, pain significantly decreases, and functionality improves within the first week [62,63]. Nevertheless, there remains controversy regarding long-term effects, which could explain some participants’ perceptions that injections do not provide lasting pain relief [64–66].

Regarding physiotherapy, the participants highlighted its role as a key component in the treatment of chronic pain and as a facilitator for recovering functionality, emphasising the importance of professional supervision during exercises to avoid undesirable effects, such as exacerbation of pain. These findings coincide with those of García et al. [67], which asserts that performing exercises under the supervision of a physiotherapist is fundamental, as there is a wide range of available interventions, and selecting the appropriate intervention for each patient is crucial for the success of treatment.

Finally, oral pharmacological treatments were perceived as ineffective, associated with adverse side effects, and limited efficacy over time. Concerning surgical treatment, the participants expressed concern or fear about undergoing this procedure, an emotion that has been widely documented by various authors [68,69]. This fear can be attributed to multiple factors, such as anaesthesia, postoperative pain, and the procedure itself. Fear arises from present threats, whereas anxiety arises from anticipation [70]. In a 2022 meta-analysis that included low/middle-income countries, one in two patients experienced preoperative anxiety attributed to a multifactorial effect [71].

### 4.1. Strengths and limitations

Our study has several methodological strengths. First, we explored the conceptualisation of patients with two different chronic pain conditions. Moreover, the focus groups were organised according to sex, which could be advantageous in distinguishing differences in access to and acceptance of treatment based on sex. Understanding patients’ opinions is essential for guiding healthcare professionals and administrators to create clinically appropriate improvements in the public health system that align with patients’ objectives.

Second, multiple techniques were employed to mitigate potential biases in the data analysis and interpretation, which are inherent in qualitative studies and could pose limitations to this study. These techniques included: (a) supervision and audit by several researchers not directly involved in analysis and interpretation; (b) independent coding, analysis, and interpretation by two researchers; and (c) a detailed description and contextualisation of the entire process for readers’ evaluation.

However, this study has limitations. Firstly, we were unable to conduct the planned six focus groups of 8–10 participants as outlined in the protocol [33]. Ultimately, five groups comprising 3–5 participants were included. This was primarily due to the participants either declining to participate, as no assessment or treatment was offered, or failing to attend the scheduled day for the focus group. Secondly, the study was conducted in a healthcare region of Lleida (Catalonia), which limits the generalization of the results; therefore, the findings should be interpreted within this context. Furthermore, the study was not designed to systematically compare subgroups (CTS vs. SAIS, gender, comorbidities), and these variables may influence perceptions, potentially revealing condition-specific patterns that were not explored. Thirdly, as a qualitative study, the results reflect the subjective perceptions and experiences of participants. These findings do not imply causal relationships, but rather offer hypotheses that could be explored in future quantitative research to assess their potential impact on clinical outcomes.

## 5. Conclusions

This study highlights the complexity of managing CMP and the need for a multidimensional approach. Strengthening the problem-solving capacity of primary health care, improving coordination between care levels, increasing human resources, personalising follow-up, and ensuring effective communication were perceived by participants as key aspects in their care experience. The facilitators and barriers identified provide relevant insights and variables that should be considered in the management of patients with CTS and SAIS, and incorporated into follow-up practices to better support patient care and experience. Future studies should evaluate the impact of these improvements on clinical and organizational outcomes.

## Supporting information

S1 File Guide focus groups. (DOCX)

## Abbreviations

CTS: Carpal Tunnel Syndrome
CMP: Chronic Musculoskeletal Pain
GP: General Practitioners
HC: Hospital Care
PHC: Primary Health Care
PHCC: Primary Health Care Centre
SAIS: Subacromial Impingement Syndrome
