# Supplementary material for: Barriers and facilitators from the patients’ perspective in the follow-up of carpal tunnel syndrome and subacromial impingement syndrome: A qualitative study
Source: PLoS One. 2026 Feb 17;21(2):e0343222. doi: 10.1371/journal.pone.0343222 (PMC12912576; doi:10.1371/journal.pone.0343222)
Supplement: S1 File — (DOCX) [file pone.0343222.s001.docx]

**Supporting Information 1. Guide for focus groups**

**1. Cordial greeting and introduction of the interviewer.**

**2. Delimit the reason for the interview and its duration:**

- Inform that the questions are designed to provide information about barriers and facilitators of treatments to enhance their acceptance.

- Inform that this is a focus group that will be recorded as indicated in the informed consent and will last approximately 30-45 minutes.

**3. Interview:**

- Can you describe the basic daily life activities that you perform?

- In which situations do you feel pain?

- What are your thoughts and feelings about pain?

- What is the most difficult aspect of the pain?

- Are you concerned about pain? Why? Do daily activities increase pain?

- Do you believe that it is safe for someone experiencing pain to be physically active?

- What has been your experience with being diagnosed with CTS or SSA?

- Who do you feel comfortable talking about regarding your physical condition?

- How often have you attended the emergency department in the last year because of physical problems?

- How did you communicate about the diagnosis, and how did you live with the illness and treatment? How are you managing?

- What aspects of treatment do you find most difficult and which do you find easier?

- How would you accept pharmacological treatment? What would this mean in terms of financial costs, time, and difficulties in your work life?

- How would you accept infiltration treatment? What would this mean in terms of financial costs, time, and difficulties in your work life?

- How would you accept the US-guided infiltration treatment? What would this mean in terms of financial costs, time, and difficulties in your work life?

- Would any of the treatments mentioned earlier cause fear, uncertainty, mistrust, or insecurity?

**Changes in Quality of Life: Before and After**

- What benefits/advantages do you expect of these treatments? Returning to work, practising sports, experiencing less pain, and social life

- How was the diagnosis communicated to you? What information were you provided with at that time?

- What information did you generally provide? Do you really know what is happening?

- Doubts: Do you have doubts? What would you like to know but do not? What do you do when you have doubts? Who do you turn to? How can you resolve these issues?

- Treatment: How has the treatment been provided by professionals?

- Understanding: Do you feel understood by the professionals?

- Support: Have you received professional support? Have you received psychological support at any time? Have you needed it?

- Healthcare: What aspects of healthcare have you changed? Which would you keep?

- Relationship with professionals: How would you define your relationships with professionals? What type of relationships would you prefer?

- Are there any aspects that you would like to add that have not been previously covered?

**4. Cordial closure and thanks:**

- Are there any aspects we haven’t covered that you would like to add?
